# Histopathological changes of the buccal mucosa and skin after botulinum neurotoxin intramuscular injection in rats (immunohistochemical study)

**DOI:** 10.1186/s12903-025-05627-w

**Published:** 2025-02-25

**Authors:** Gihan S. Hassan, Basant H. Abouzaid, Reda G. Saleh

**Affiliations:** 1 Oral Biology Department, Faculty of Dentistry, Tanta University, Tanta, Egypt; 2 Oral Pathology Department, Faculty of Dentistry, Tanta University, Tanta, Egypt

**Keywords:** Apoptosis, Botulinum neurotoxin type A, Buccal mucosa, Caspase 3, Degeneration, Skin

## Abstract

**Background:**

A surplus of clinical studies focused mainly on the clinical impacts of botulinum neurotoxin type A disregarding the histopathological changes stemming from its injection. This study was designed to inspect the potential effects of botulinum neurotoxin type A injection on the skin and buccal mucosa in both the injected and non-injected sides after one week and one month as no available studies have addressed its histopathological effects.

**Methods:**

Twelve rats were injected with a single injection of 2.5 U of botulinum neurotoxin type A intramuscularly near the angle of the mouth on the right side. After one week, six injected rats were euthanized, and tissue samples were obtained from both the injection (*n* = 6) and the non-injection (*n* = 6) sides. After 4 weeks, six injected rats were euthanized, and tissue samples were obtained from both the injection (n = 6) and the non-injection (*n* = 6) sides. Six rats served as non-injected controls (*n* = 6). Samples were prepared for histological and immunohistochemical examination with caspase-3.

**Results:**

Caspase-3 expression showed a highly significant increase (*P* = 0.005) in staining intensity in specimens of one week as compared to one month groups. At the non-injected side, the overall caspase-3 immunostaining score showed a significant elevation (*P* = 0.03) after one-month of botulinum neurotoxin type A injection.

**Conclusion:**

Botulinum neurotoxin type A injection was associated with degenerative changes in the buccal mucosa and skin, nevertheless these changes declined after one month in the injected side. More degenerative changes were depicted in the non-injected side and increased after one month. Therefore, botulinum neurotoxin type A injection may affect not only the patient’s needs and expectations but also their health.

## Background

Botulinum neurotoxins (BoNTs) are proteins produced by *Clostridium Botulinum* bacteria which are considered extremely potent neurotoxins. BoNTs affect the peripheral cholinergic neurons. They effectively prevent the release of acetylcholine, a neurotransmitter that activates glandular secretion and the contraction of muscles, leading to hyposecretion of glands and profound, albeit transient neuromuscular paralysis [1]. Multiple serotypes of BoNTs are found namely, A-G, that vary in biogenesis, molecular size, and mechanism of action. The most effective and frequently used serotype is Type A (BoNT-A) [2].

BoNT-A is not only used in cosmetic conditions but also in therapeutic conditions such as blepharospasm, cervical dystonia, strabismus, hemifacial spasm, severe primary axillary hyperhidrosis, glabellar wrinkles tremor, hemifacial spasm, hyperhidrosis, and headache [3, 4]. In dentistry, it is used in sialorrhea, bruxism, mandibular spasm, gummy smile, oromandibular dystonia, masseteric hypertrophy, trigeminal neuralgia, and temporomandibular joint disorders [5].

Despite some local effects at the site of injection such as pain, ecchymosis, swelling, and hypesthesia, BoNT-A is generally regarded as a safe medication; lacking serious side effects [5]. Many studies reported its effects on various tissues and organs. Suh et al. [6] illustrated ultrastructural changes in the myotendinous nerve endings after BoNT-A injection into the cat extraocular muscle. Lee et al. [7] depicted disturbance in eye alignment in 4% of patients with essential blepharospasm after BoNT-A injection. Also, BoNT-A injection led to nasal gland apoptosis in guinea pigs [8].

It is well-established that the process of apoptosis involves intrinsic and extrinsic pathways which both encompass the cascade activation of caspases. Caspase-3 is one of the executing caspases which is important for cell death. Many caspase substrates are cleaved by caspase-3. It can process pro-caspase 2, 6, 7, and 9 which illustrates the extensive involvement of caspase-3 in the process of cell death. In addition, caspase-3 is linked to some of the changes in cell morphology (membrane blebbing, DNA fragmentation, chromatin condensation) and biochemical events associated with the execution and completion of apoptosis [9]. Moreover, it was reported that BoNT-A was able to promote caspase-3-dependent apoptotic pathways in breast cancer cell lines [10].

Noteworthy, a surplus of clinical studies focused mainly on BoNT-A’s clinical impacts disregarding the histopathological changes stemming from its injection. Considering that BoNT-A is frequently used to treat functional dental along with aesthetic conditions, particularly in the elimination of facial wrinkles, contouring of the lower face, gummy smile, and facial asymmetry [5, 11]. Therefore, it was mandatory to study the histopathological effects of BoNT-A injection on the skin and mucosa of the cheeks at the perioral area. The current study was performed to resolve the following points: (1) The effects of BoNT-A injection on the epithelial cells of the buccal mucosa and skin and the cells of the underlying connective tissue at the area of injection after one week. (2) Analyzing the degrees of tissue injury through the inspection of the signs of pathological changes histologically. (3) The detection of the apoptotic changes immunohistochemically through caspase-3 expression in these tissues. (4) The distant effects of BoNT-A injection at the other side of the mouth. (5) Recognizing if these impacts declined or even disappeared after one month at both the injected and non-injected side.

## Materials and Methods

### Animal model

All animal procedures were approved by the Institutional Committee of Ethics at Faculty of Dentistry, Tanta University (#R-OB-9–22-8) and were conducted in accordance with the guidelines laid down by the ARRIVE (Animal Research: Reporting In Vivo Experiments) guidelines for conducting animal research. Eighteen healthy adult male Albino rats (200–300 g) were used in this study. The animals were served in the animal house of the Histology Department, Faculty of Medicine, Tanta University. They were kept in polypropylene cages; five animals in each cage, at a temperature of 23 ± 2 °C and a day/night cycle of 12 h. They received a diet of standardized pellets and free access to tap water.

### Chemicals

BoNT-A (Refinex 50U—KC Pharmaceuticals. www.refinex-jp.com) was reconstituted in 1 mL of 0.9% sodium chloride solution (saline). A syringe for BoNT-A residue zero, ultra‐fine with 0.5 mL (50 UI) was used for BoNT-A injections. BoNT-A injection given to each animal was adjusted to a dose of 2.5 U [12]. Anesthetic injections were performed with ketamine (10 mg/kg body weight -Ketalar, Pfizer, Turkey) and xylazine hydrochloride (0.5 mg/kg body weight- Rompun, Bayer), by an insulin needle. Anti-Caspase-3 antibody (Rabbit monoclonal [EPR18297] to Caspase-3, Abcam, ab184787) was used for immunohistochemical examination.

### Experimental design

Rats (*n* = 18) were divided randomly as follows: twelve rats were injected with a single intramuscular injection of 2.5 U of BoNT-A near the angle of the mouth in the right-side. After 1 week, six injected rats were euthanized and sacrificed. Then tissue samples were obtained from the injection side (*n* = 6) and from the non-injection side (*n* = 6). After 4 weeks, six injected rats were euthanized and sacrificed. Then tissue samples were obtained from the injection side (*n* = 6) and from the non-injection side (*n* = 6). Six rats served as non-injected controls (*n* = 6). For all injections, animals were anesthetized intramuscularly with ketamine and xylazine hydrochloride. The site of injection was shaved and disinfected with chlorhexidine. The point of injection was set at 5 mm on a straight horizontal line from the angle of the mouth.

### Animal euthanasia and samples processing

After anesthesia, rats were euthanized by cervical dislocation to minimize pain and discomfort. Then, the site of injection was shaved for any remnants of hair and cleaned with chlorhexidine. The skin and buccal mucosa were immobilized by applying light pressure with the fingers of one hand. A metal spatula was placed under the buccal mucosa for support. A circular punch was performed with the other hand by a sterile 10 mm diameter punch biopsy[13]. Thus, the removed specimen included both the skin and buccal mucosa. Two specimens were obtained from each animal, one representative of the oral and skin tissues at the injection side and the other comprised of comparable tissues at the non-injected side. The sites of injections at the right side and the same site of the other left half were removed containing both skin and buccal sides for histological examination.

### Histopathological study

Each specimen was fixed in a 10% formalin solution, embedded in paraffin blocks, and cut into two 4-µm-thick sections by rotary microtome. One section was routinely stained for hematoxylin and eosin (H&E) [14] while the other section was dewaxed in xylene and rehydrated then underwent antigen retrieval in a preheated water bath under citrate buffer (pH 6). Endogenous peroxidase activity was blocked via incubating sections with hydrogen peroxide, then sections were immunohistochemically stained against caspase-3 monoclonal antibody according to manufacturer protocol. The immune reaction was demonstrated by adding DAB solution (3,3´-Diaminobenzidine). For evaluation of immunostaining, any brown pigment within the nucleus and/or cytoplasm of epithelial cells was considered a positive reaction. Several parameters were employed for the assessment of caspase-3 expression:

- Overall staining intensity: measured using the grading scale (negative, weak, moderate, and strong).
- Type of stained cells: basal and/or parabasal and diffuse.
- Pattern of staining: nuclear, cytoplasmic, and nucleocytoplasmic.
- Percentage of positive cells: assessed by ImageJ software according to the method modified from Varghese 2014 [15].

To determine the percentage of positive cells and fraction of cells that are high positive, low positive, or positive in each slide, image analysis software; the ImageJ (Public domain, image processing and analysis in Java, http://rsb.info.nih.gov/ij/) was used. The following steps were performed for rat skin as well as mucosal tissues separately.

1. Each slide was scanned at low magnification (×100) to determine sites of caspase 3 positivity within epithelial cells.
2. From each slide, a minimum of 3 arbitrarily selected microscopic fields of caspase-3 immunoreactivity at the same magnification (×400) were photographed.
3. The software was then calibrated to magnification of the image (Global scale of the image analysis was set as 868 pixels = 1 µm, in a pixel ratio of 1).
4. Images were opened in ImageJ, followed by deconvolution using the optimized color deconvolution ‘IHC profiler’ plugin.
5. With the selection of the ‘H DAB’ vector on the color deconvolution popup window, IHC profiler automatically plotted a histogram profile of the DAB image, and the corresponding scoring log was displayed on the screen.
6. Then, percentages of high positive, low positive, and positive cells were checked in the scoring log and the average for the three images was calculated.
7. For additionally calculating the H-score, we used the equation: H-score = 1 % of mildly stained cells ) average of positive cells scored by ImageJ) + 2 % of moderately stained cells (average of the low positive cells scored by ImageJ) + 3 % of strongly stained cells (average of the high positive cells scored by ImageJ) [16].

### Analysis of epithelial changes

Light microscope (Leica DM500) equipped with a built-in camera (Leica ICC50 HD Camera system) was used. H&E-stained tissue sections were examined for signs of pathological changes. Variable degrees of tissue injury were observed within the epithelium and the underlying connective tissue. Accordingly, the following parameters were considered for assessment: cellular swelling, increased cytoplasmic eosinophilia, vacuolar degeneration, and nuclear changes such as pyknosis, faded chromatin (karyolysis), fragmented or disappeared nuclei [17]. Each change was scored on a binary basis (0, no change; 1, detectable change) then, the mean score was calculated for each group. The underlying connective tissue was observed for signs of fibrosis or muscle atrophy.

### Statistical analysis

Images of anti-Caspase-3 immunostained sections were analyzed using the ImageJ analysis system (ImageJ 1.48 s, Wayne Rasband, National Institute of Health, USA). Statistical analysis was performed using IBM SPSS statistics for Windows, Version 26 (Armonk, NY: IBM Corp.). The Chi-square test was used to assess the differential expression of Caspase-3. The overall H-score was evaluated via a one-way ANOVA test. Subsequent comparisons between groups were done by a Tukey test. In addition, a paired t-test was employed to evaluate H-score results in all groups of BoNT-A injection. Degenerative changes were presented as a mean ± standard deviation (SD). P value ≤ 0.05 was considered significant.

## Results

### Histopathological findings

#### Control Group (*n* = 6)

**Fig. 1:**
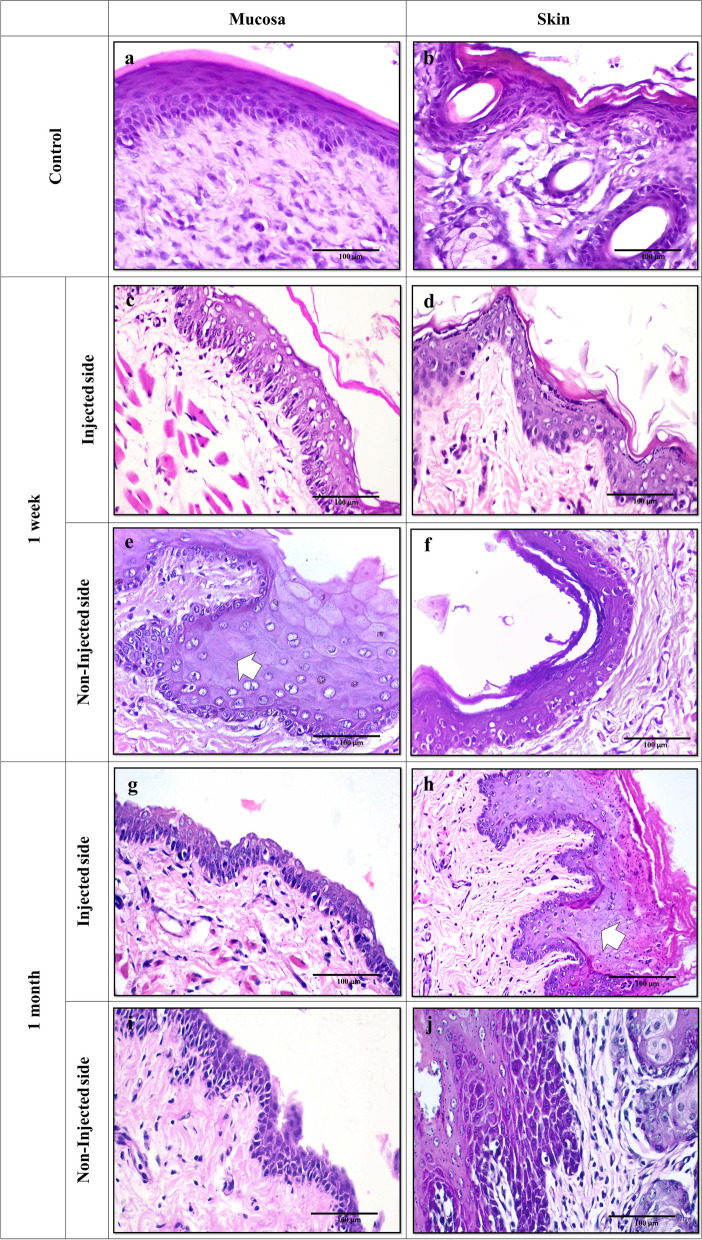
(**a**) Normal rat mucosa showing orthokeratinized stratified squamous epithelium with normal stratification. The underlying connective tissue consists of coarse collagen fibers, plump fibroblasts, and fine capillaries with no signs of inflammation. (**b**) Normal rat skin tissue showing thin orthokeratinized stratified squamous epithelium harboring several hair follicles (H&E; original magnification (**a**) X40, (**b**) X20). (**c** – **f**) Rat tissues after one-week BoNT-A injection. (**c**) At the injected side, buccal mucosa exhibits atrophic epithelium with a thin, detached layer of orthokeratin. Numerous vacuolated cells are seen and disorganized muscles in connective tissue. (**d**) The skin on the injected side shows numerous vacuolated cells. (**e**) On the non-injected side, buccal mucosa shows cells with washed-out chromatin pattern and cellular swelling with inconspicuous nuclei (arrows). (**f**) Rat skin at the non-injected side shows increased numbers of cells with pyknotic nuclei and shrunken cytoplasm at basal and parabasal layers. (**g** – **j**) Rat tissues after one-month BoNT-A injection. (**g**) Buccal mucosa at the injected side shows scattered vacuolated cells with pyknotic nuclei. (**h**) The skin at the injected side exhibits thickened epithelium with prickle cells showing signs of swelling and disappearing nuclei (arrow). (**i**) At the non-injected side, buccal mucosa shows atrophic epithelium with numerous cells displaying pyknotic nuclei with clumped chromatin and shrunken cytoplasm. (**j**) Rat skin at the non-injected side shows prominent cytoplasmic eosinophilia with predominantly clumped chromatin and disordered stratification (H&E; original magnification (**a** – **j**) X40)

The normal rat buccal mucosa revealed regular oral mucosal epithelium exhibiting a normal maturation pattern with an average thickness of 10 – 12 cells thick. A well-defined basal cell layer was observed. The overlying prickle cell layer showed ample eosinophilic cytoplasm and vesicular nuclei. A superficial layer of ortho-keratin was noticed with a prominent underlying granular cell layer. The connective tissue showed collagen fibrils, blood capillaries, and scattered plump fibroblasts. Numerous aggregates of skeletal muscle tissue were evenly distributed throughout the lamina propria and consisted of moderate-sized muscle cells with rounded, vesicular nuclei (Fig. 1.a). Likewise, the nearby rat epidermis showed ortho-keratinized, stratified squamous epithelium of even thickness with numerous hair follicles. The underlying dermis exhibited a similar appearance to that of the intra-oral mucosa except for variable numbers of skin adnexal structures (hair, sweat glands, and sebaceous glands) (Fig. 1.b).

#### One-week BoNT-A (*n* = 6)

Examination of the rat buccal mucosa at the injection side revealed several tissue changes. The stratified squamous epithelium showed an average thickness of 5 – 10 cells with a generally thin, detached ortho-keratin layer. The basal cell layer exhibited disrupted arrangement in many areas of the lining. Moreover, the prickle cell layer showed enlarged vesicular nuclei with prominent nucleoli and enhanced cytoplasmic eosinophilia. Numerous cells demonstrated signs of cellular degeneration in the form of vacuolated cytoplasm, clumped chromatin, and nuclear pyknosis (Fig. 1.c). The average degeneration score was 3.6 ± 0.8. Whereas the adjacent skin epithelium exhibited 4 – 8 layers thick with a thin ortho-keratinized surface (Fig. 1.d). The average degeneration score was 2 ± 1.2. The connective tissue showed haphazardly arranged collagen fibers with plump fibroblasts and scattered mast cells. The underlying muscle layer exhibited signs of atrophy with irregular, loose bundles, slender and fragmented muscle fibers (Fig. 1.c). One specimen exhibited areas of neutrophilic infiltration within the connective tissue associated with secondary bacterial infection. Whereas the skin and buccal mucosa on the non-injected side exhibited similar histopathologic changes with a mean degeneration score 3.6 ± 0.57 (Fig. 1. e and f).

#### One-month BoNT-A (*n* =6)

At the injection side, the rat buccal mucosa showed overall atrophic stratified squamous epithelium ranging in thickness from 4 – 8 cells. The overlying ortho-keratin appeared thin and discontinuous. The basal cell layer appeared more organized, yet with vague palisading. The prickle cell layer showed enlarged nuclei with dispersed chromatin and prominent nucleoli. Some cells showed darkly stained, hyperchromatic nuclei and enhanced cytoplasmic eosinophilia. Scattered cells showed signs of degeneration in the form of shrunken cytoplasm with clumped chromatin and perinuclear clear halo. Areas of intraepithelial clefting and separation were also seen. The underlying connective tissue showed atrophied muscle fibers and numerous mast cells (Fig. 1.g). Comparably, the histopathologic changes observed in the rat skin at the injected side appeared to be more pronounced. More prickle cells showing washed-out chromatin pattern were detected. Cells with pyknotic nuclei and shrunken cytoplasm were more numerous in the basal and parabasal layers. Moreover, other signs of cellular degeneration as cellular swelling and faded chromatin pattern (karyolysis) were noticed (Fig. 1.h). The average degeneration score was 2.6 ± 0.89 for skin and buccal mucosa. Similarly, the changes on the non-injected side appeared to be more augmented (Fig. 1.i and j). The average degeneration score was 3.5 ± 0.57 and 3 ± 1 for buccal mucosa and adjacent skin, respectively.

### Immunohistochemical findings

**Fig. 2:**
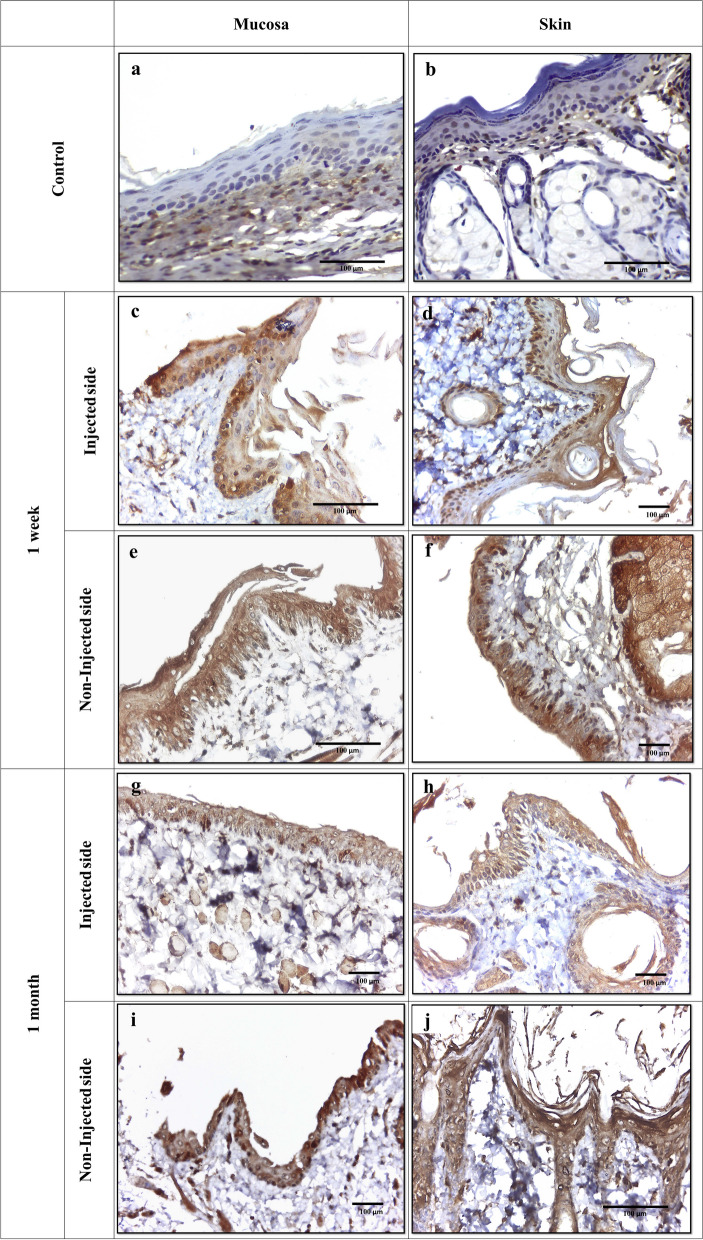
Immunohistochemical staining of rat tissues with anti-caspase-3. Control tissue shows negative expression of caspase-3 in rat epithelial cells in both oral mucosa (**a**) and skin (**b**). Positive immunostaining is observed in the cytoplasm of connective tissue fibroblasts. After a one-week BoNT-A injection, positive nuclear and cytoplasmic caspase-3 expression was predominantly detected in basal and parabasal layers of rat mucosa (**c**) and skin (**d**) at the injected side. At the non-injected side, enhanced nucleocytoplasmic caspase-3 immunostaining is noticed in all epithelial layers in mucosa (**e**) and skin (**f**). The group that received one-month BoNT-A injections shows moderate diffuse, nucleocytoplasmic caspase-3 expression in the mucosa (**g**) and skin (**h**) at the injected side. The tissues at the non-injected side exhibit enhanced diffuse, nucleocytoplasmic caspase-3 expression in mucosa (**i**) and skin (**j**). A positive reaction is evident within connective tissue fibroblasts in all specimens (DAB; original magnification (**c**), (**e**), (**j**) X40, (**d**), (**f**), and (**g** – **i**) X20)

Considerable variation regarding the pattern of caspase-3 staining was evident among the study groups. The control group showed negative caspase-3 expression. The underlying connective tissue fibroblasts, sebaceous cells, and hair follicle epithelial cells showed varied consistently caspase-3 positive expression [18]. Conversely, positive caspase-3 immunostaining was noticed in the oral and dermal epithelial cells as well as sebaceous cells and fibroblasts in all study groups in the form of nuclear, cytoplasmic, or combined nucleocytoplasmic expression. Of note, a highly significant upregulation in the staining intensity of caspase-3 was observed in the oral mucosal epithelium in specimens of injected side at one week as compared to those of one-month duration (*P* = 0.005). Whereas it was the opposite in non-injected sides (Table 1) (Fig. 2). In addition, the distribution of caspase-3 immunostaining showed highly significant difference (*P* = 0.001) within oral epithelia since all specimens of one-month duration showed diffuse staining compared to predominant basal/parabasal expression in some specimens in the one-week group (Fig. 2 c and d). Likewise, the epidermis showed a significant difference in caspase-3 distribution among groups (*P* = 0.04). Furthermore, nucleocytoplasmic staining was significantly enhanced (*P* = 0.003) in the epidermal cells of rats after one-week and one-month BoNT-A injections compared to other subcellular localizations (Table 2).

**Table 1:** Comparison between the studied groups regarding Caspase-3 expression in the oral mucosa

**Parameters**		Control group *N* = 6		Injected side *N* = 6		Non injected side *N* = 6		*p*-value
		**1st week**	**4th week**	**1st week**	**4th week**	**1st week**	**4th week**	
		**N (%)**	**N (%)**	**N (%)**	**N (%)**	**N (%)**	**N (%)**	
**Expression**	Positive	0(0%)	0(0%)	6(100%)	6(100%)	5(83.3%)	6(100%)	3.130 (0.372)
	Negative	0(0%)	0(0%)	0(0%)	0(0%)	1(16.7%)	0(0%)	
**Staining intensity**	Mild	0(0%)	0(0%)	0(0%)	0(0%)	2(40%)	0(0%)	18.338 (0.005*)
	Moderate	0(0%)	0(0%)	6(100%)	1(16.7%)	2(40%)	2(33.3%)	
	Strong	0(0%)	0(0%)	0(0%)	5(83.3%)	1(20%)	4(66.7%)	
**Type of stained cells**	Basal and parabasal	0(0%)	0(0%)	2(33.3%)	0(0%)	5(100%)	0(0%)	16.702 (0.001*)
	Diffuse	0(0%)	0(0%)	4(66.7%)	6(100%)	0(0%)	6(100%)	
**Pattern**	Cytoplasmic	0(0%)	0(0%)	1(16.7%)	0(0%)	0(0%)	0(0%)	20.152 (0.003*)
	Nuclear	0(0%)	0(0%)	2(33.3%)	0(0%)	5(100%)	0(0%)	
	Nucleocytoplasmic	0(0%)	0(0%)	3(50%)	6(100%)	0(0%)	6(100%)

**Table 2:** Comparison between the studied groups regarding Caspase-3 expression in the skin

**Parameters**		Control group *N* = 6		Injection side *N* = 6		Non injection side *N* = 6		*p*-value
		**1st week**	**4th week**	**1st week**	**4th week**	**1st week**	**4th week**	
		**N (%)**	**N (%)**	**N (%)**	**N (%)**	**N (%)**	**N (%)**	
**Expression**	Positive	0(0%)	0(0%)	6(100%)	6(100%)	5(83.3%)	6(100%)	3.130 (0.372)
	Negative	0(0%)	0(0%)	0(0%)	0(0%)	1(16.7%)	0(0%)	
**Staining intensity**	Mild	0(0%)	0(0%)	1(16.7%)	0(0%)	1(20%)	0(0%)	10.569 (0.103)
	Moderate	0(0%)	0(0%)	5(83.3%)	2(33.3%)	4(80%)	3(50%)	
	Strong	0(0%)	0(0%)	0(0%)	4(66.7%)	0(0%)	3(50%)	
**Type of stained cells**	Basal and parabasal	0(0%)	0(0%)	2(33.3%)	0(0%)	3(60%)	0(0%)	8.110 (0.044*)
	Diffuse	0(0%)	0(0%)	4(66.7%)	6(100%)	2(40%)	6(100%)	
**Pattern**	Cytoplasmic	0(0%)	0(0%)	0(0%)	2(33.3%)	0(0%)	4(66.7%)	20.225 (0.003*)
	Nuclear	0(0%)	0(0%)	0(0%)	0(0%)	3(60%)	0(0%)	
	Nucleocytoplasmic	0(0%)	0(0%)	6(100%)	4(66.7%)	2(40%)	2(33.3%)	

**Fig. 3:**
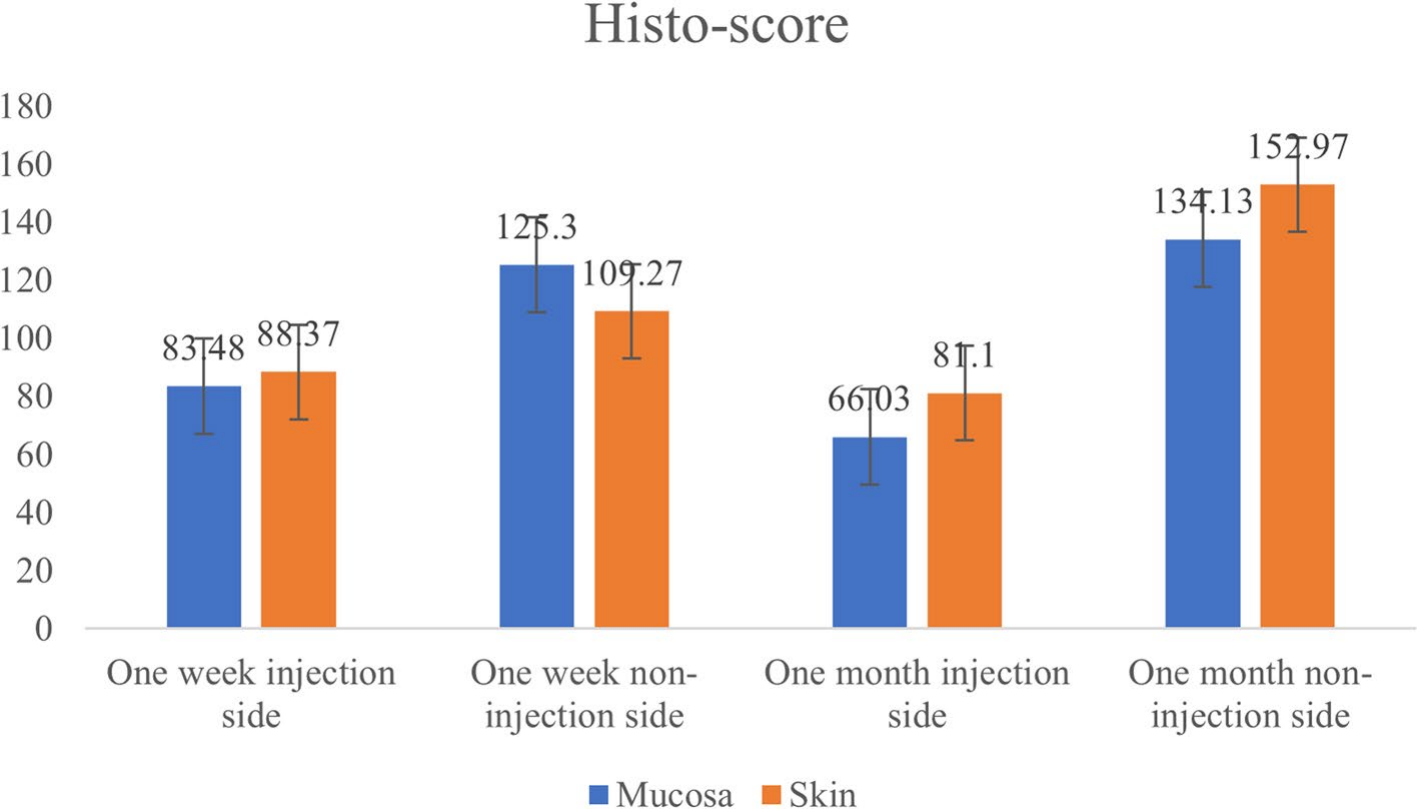
Bar chart illustrates the total H-score of caspase-3 immunostaining in different study groups, (*N* = 6)

The H-score displayed remarkable fluctuations in caspase-3 expression with respect to increased duration after BoNT-A injections (Fig. 3). At the injected side, the oral mucosa exhibited a mean H-score of 83.48 ± 21.04 at one-week duration which declined to 66.03 ± 39.89 after one month. Similar scores were noticed in the skin specimens. On the other hand, at the non-injected side, H-score values showed noticeable elevation after one month of BoNT-A injection, which was revealed to be statistically significant (*P* = 0.03).

## Discussion

BoNT-A is a potent toxin derived from the bacterium *Clostridium botulinum* that has been widely used in several therapeutic as well as cosmetic settings [19]. BoNT-A injections showed efficacy in the treatment of disorders involving abnormal muscle function since it blocks the release of acetylcholine in the neuromuscular junction with subsequent reversible muscle relaxation [20]. In dentistry, it is used to treat functional dental along with aesthetic conditions as to smoothen wrinkles around the mouth [11] and wrinkles produced by the muscles of facial expression such as deep nasolabial folds [5]. Accordingly, the perioral area near the angle of the mouth was selected for BoNT-A injection in the current study.

After one week of BoNT-A injection, the buccal mucosa and adjacent skin demonstrated atrophic epithelium and degenerative changes. These alterations were similar to the findings of Chuang et al. (2006) [21] who injected rat prostate with BoNT-A. The authors observed diffuse atrophy of the prostate, flattening of their epithelial cells, increased number of apoptotic cells with diminished cellular proliferation and α_1A_-adrenoceptor suppression. These effects disappeared after two weeks. They related these changes to the inhibitory effect of BoNT-A on cholinoceptors which could modulate cellular functions such as mitosis, proliferation as well as differentiation [22].

The prevalence of cellular degeneration within the epidermal cells in our study would stem from the notion that the epidermal keratinocytes usually express one or more of the BoNT-A-binding proteins SV2, FGFR3, or vanilloid receptors and/or BoNT-A-cleavage target SNAP-25. Likewise, the degenerative effects on mucosal keratinocytes would be attributed to the ability of BoNT-A to cleave SNAP-23 which is widely expressed in mucosal tissues [23]. After binding, the exact mechanism by which BoNT-A mediates its effects is questionable. It has been suggested that BoNT-A disrupts the intercellular epithelial barrier either via binding the E-cadherin molecule [24] or through affecting cell morphology and viability [25]. Furthermore, the similar tissue changes we observed at the non-injected side could reflect the diffusion ability of BoNT-A since it can disperse 30 – 45 mm away from injected muscles even if fasciae were present [19].

Of note, our study revealed that the skin and mucosa on the non-injected side exhibited a higher degree of cellular degeneration in the one-week and one-month groups. Such a remote effect on non-injected tissues would be linked to the potential diffusion of the drug through the circulation, a phenomenon supported by the findings of Hristova et al. (2012) [26]. They reported that BoNT-A can permeate through intact vessel walls in addition to the possibility of inadvertent intravenous injection. Another potential pathway for BoNT-A spread is the retrograde transport through microtubules in the nerve axon to motor and sensory regions in the spinal cord [27]. A combination of vascular dissemination and retrograde axonal spread of BoNT-A is probably responsible for the distant side effects [28].

Additionally, the increased degeneration scores at the non-injected sides in one-week and one-month groups would be plausible according to the findings of Ravichandran et al. (2013) [29] who demonstrated that blood had no impact on the structure, function, or biological half-life of BoNT-A. Whereas the drug at the injection side may be diluted or negatively affected by the local allergic reactions (edema, erythema, and urticaria) known to develop at the sites of BoNT-A injection [19]. The latter would explain our finding regarding the existence of mast cells primarily in tissues at the injection side. In the same context, the results of the present study revealed decreased epithelial thickness after BoNT-A administration as compared to the control group. This was in line with the observations of Ward et al. (2012) [30] who reported decreased acanthosis in a mouse model of psoriasiform dermatitis after BoNT-A injection.

Furthermore, our findings revealed that BoNT-A effects extended to involve the underlying connective tissue and associated musculature which displayed atrophic signs along with increased fibrosis. The primary therapeutic effect of BoNT-A is relieving muscle contractions via chemical denervation due to the blockade of acetylcholine release [23]. As a consequence, muscles became unstimulated, hence it is not uncommon to see microscopic signs of atrophy in a manner comparable to disuse atrophy [31]. Additionally, Pingel et al. (2017) [32] related the enhanced synthesis of collagen to the acceleration of tissue remodeling following muscular destruction.

Apoptosis is a highly regulated biochemical process, essential for the maintenance of tissue homeostasis and the elimination of unwanted cells. It is manifested as a group of morphological cellular changes mediated by a family of cysteine proteases called Caspases. Caspase-3 is one of the effector Caspases that, once activated by the initiator Caspases, can cleave cytoplasmic and nuclear proteins [33], thus mediating the dramatic signifying apoptosis [34]. Therefore, caspase 3 was used to investigate the apoptotic changes in these tissues in the current study.

Our results showed positive Caspase-3 immunostaining within the epithelial cells as well as connective tissue fibroblasts in all the study groups except for the control group. Increased apoptosis in the epithelial cells is in accordance with the potential negative effects of BoNT-A on the intercellular bridges or the cell viability discussed before. Moreover, BoNT-A can directly affect the dermal fibroblasts in several ways. BoNT-A inhibits fibroblast proliferation, stops fibroblasts–to–myofibroblasts differentiation via counteracting transforming growth factor-β, and induces apoptosis [23, 35].

In addition, the nucleocytoplasmic localization of Caspase-3 immunostaining was revealed to be prevalent among BoNT-A groups. This would confirm the active apoptosis process since Caspase-3 exists in the cytoplasm in a precursor form then it undergoes translocation from the cytoplasm to the nucleus following apoptosis initiation due to disruption of nuclear-cytoplasmic barrier [34]. In the same context, the significant increase in Caspase-3 staining intensity within one-month group (*P* = 0.005) coined with the diffuse staining within all epithelial layers (*P* = 0.001) as compared to decreased intensity and predominant basal and parabasal expression in one-week group would highlight the cumulative effects of BoNT-A that usually reaches a peak of maximum effects within 1 – 4 weeks after injection. It is noteworthy that the average of BoNT-A effects declines through three months and the prolongation of its effects requires repeated injections [20]. This coincided with our H-score results which showed some decline after one month of BoNT-A injection at the skin and mucosa on the injected side. Whereas on the non-injected side, the H-score exhibited opposite results which agreed with our explanation of the increased accumulative effect of BoNT-A shown by increased caspase-3 immune intensity of this group.

In conclusion, after one week of BoNT-A injection, the buccal mucosa and the adjacent skin demonstrated atrophic and degenerative changes that showed a slight decrease after one month in the injected side. At the non-injected side, the degeneration and cellular apoptosis were more significant than at the injected side and showed a slight increase after one month in both skin and mucosa. Consequently, in the clinical determination, the excessive usage of BoNT-A should be reviewed since its effects are not constrained to the injected side. In addition, the current study has some limitations including a single dose with a specific volume, the use of one marker, and short experimental periods. Therefore, further studies with a larger sample size, longer experimental periods and using more immunohistochemical markers are necessary to verify, explain and clarify these potential effects of BoNT-A.

## Data Availability

All data are available from the corresponding author upon a reasonable request.
